# ZIF-8 Nanoparticles Based Electrochemical Sensor for Non-Enzymatic Creatinine Detection

**DOI:** 10.3390/membranes12020159

**Published:** 2022-01-28

**Authors:** Titisha Chakraborty, Munmun Das, Chan-Yu Lin, Yen Su, Bing Yuan, Chyuan-Haur Kao

**Affiliations:** 1 Department of Electronic Engineering, Chang Gung University, 259 Wenhwa 1st Road, Guishan District, Taoyuan 33302, Taiwan; titisha.1987@gmail.com (T.C.); munmundas221987@gmail.com (M.D.); tp963332@gmail.com (Y.S.); chenguan052@gmail.com (B.Y.); 2 Kidney Research Center, Department of Nephrology, Chang Gung Memorial Hospital, No.5, Fuxing St., Guishan District, Taoyuan 33302, Taiwan; r2534@cgmh.org.tw; 3 Department of Electronic Engineering, Ming Chi University of Technology, 284 Gungjuan Rd., Taishan District, New Taipei City 24301, Taiwan; 4 Center for Green Technology, Chang Gung University, 259 Wenhwa 1st Road, Guishan District, Taoyuan 33302, Taiwan

**Keywords:** ZIF-8 NPs, non-enzymatic, creatinine, sensor, creatinine-Zn composite

## Abstract

There is a consistent demand for developing highly sensitive, stable, cost-effective, and easy-to-fabricate creatinine sensors as creatinine is a reliable indicator of kidney and muscle-related disorders. Herein, we reported a highly sensitive and selective non-enzymatic electrochemical creatinine sensor via modifying poly(3,4 ethylenedioxythiophene) polystyrene sulfonate (PEDOT:PSS) coated indium tin oxide (ITO) substrate by zeolitic imidazolate framework-8 nanoparticles (ZIF-8 NPs). The topography, crystallinity, and composition of the sensing electrode were characterized by field emission scanning electron microscopy (FESEM), atomic force microscopy (AFM), X-ray diffraction (XRD), and X-ray photoelectron spectroscopy (XPS). The peroxidase-like activity of ZIF-8 nanoparticles enabled it to detect creatinine forming a zinc-creatinine composite. The electrochemical behavior and sensing performance were evaluated by amperometric and impedimetric analysis. The sensor obtained a sufficiently low limit of detection (LOD) of 30 µM in a clinically acceptable linear range (0.05 mM–2.5 mM). The interference study demonstrated high selectivity of the sensor for creatinine concerning other similar biomolecules. The sensing performance of the creatinine sensor was verified in the actual human serum, which showed excellent recovery rates. Hence, the magnificent performance of ZIF-8 based non-enzymatic creatinine sensor validated it as a responsible entity for other complicated renal markers detection.

## 1. Introduction

Creatine, a source of energy in the form of phosphate, can be found in the muscles of a healthy individual [1]. Creatine produces a nitrogenous waste called creatinine that is excreted through the kidneys by glomerular filtration [2]. The standard clinical range of serum creatinine is 40–150 µM, which is approximately constant for healthy persons but can rise beyond 1000 µM due to illness [3]. The creatinine level other than the typical normal range is a sign of several fatal diseases such as chronic kidney disease (CKD), cardiovascular diseases, muscular disorders, and Parkinson’s [4]. Although there are several renal failure markers for renal failure, including urine protein, lipocalin 2, and cystatin C, creatinine is granted as the primary one as an excellent clearance level in urine, interpretative simplicity, and high susceptibility [5]. Hence, early quantitative detection of serum creatinine can resist the cumulative progression of kidney-related diseases. 

Electrochemical and optical creatinine sensors are already reported in various studies. The optical detection involved a chromogen produced by Jaffe reaction between picric acid and creatinine in an alkaline medium, but the influences of interfering chromogens are unavoidable [6]. Hence, the main challenge of this method is the lower specificity for creatinine. In contrast, an electrochemical sensor is preferred to detect creatinine over other analytical methods as it uses electron transfer for signal accession. Consequently, the electrochemical technique results in the miniaturization of the sensing device, faster response time, low fabrication expense, excellent sensitivity, improved specificity, and selectivity [7]. In order to obtain the selective detection of creatinine, electrochemical sensors are developed by immobilizing enzymes targeting creatinine molecules utilizing the detection of hydrogen peroxide (H_2_O_2_) produced during the enzymatic reaction [8]. However, the poor stability for long-term storage, severe experimental conditions, interfering byproducts, the complex synthesis procedure of the enzymatic creatinine sensor result in the requirement of developing non-enzymatic electrochemical creatinine sensors to overcome the drawbacks mentioned above [9].

Metal ions including nickel (II), copper (II), cadmium (II), mercury (II), cobalt (II), and zinc (II) have been utilized as they can directly sense creatinine involving their donor groups [10]. These metal ions can form complexes with creatinine which possesses peroxidase-like activities. However, the metal–organic (MOF) frameworks replace traditional metals and metal nanostructures with enhanced peroxidase mimicking properties. Metal–organic frameworks are basically nanoporous structures with metal ions as the connectors and organic ligands as linkers [11]. MOFs have drawn attention as peroxidase mimicking agents due to their definite pore structure, extensive surface area, flexible chemical composition, and excellent catalytic activities [12]. Zeolitic imidazolate framework-8 (ZIF-8) nanostructures, composed of Zn^2+^ ions connected by imidazolate linkers, are a particular MOF structure with zeolite type structure [13]. ZIF-8 comprises a large pore opening of 11.6 Å, enhanced surface area of 1413 m^2^/g, good thermal stability up to 550 °C, and exceptional chemical stability [14]. The attractive properties of ZIF-8 mentioned above facilitate it in the applications such as gas separation, sensing, and catalysis [15]. Moreover, the Zn^2+^ ions in a ZIF-8 molecule can form a Zn-creatinine complex on the ZIF-8 based electrodes [16]. This property of ZIF-8 can be utilized for the quantitative determination of creatinine. 

In this report, we have developed a ZIF-8/PEDOT:PSS/ITO based non-enzymatic creatinine sensor for the first time to the best of our knowledge. The topography, crystallinity, and composition of the solution-prepared ZIF-8 were characterized by field emission scanning electron microscopy (FESEM), atomic force microscopy (AFM), X-ray diffraction (XRD), and X-ray photoelectron spectroscopy (XPS). The modification steps involved for electrode fabrication were determined by cyclic voltammogram (CV) and electrochemical impedance spectroscopy (EIS) measurement, whereas the quantification of creatinine in the analyte was performed by EIS measurement. The feasibility test for the creatinine sensor was conducted by employing the device in the actual human serum spiked with different creatinine concentrations. 

## 2. Materials and Methods

### 2.1. Chemicals and Reagents

Zinc nitrate hexahydrate (Zn(NO_3_)_2_, 6H_2_O), 2-methylimidazole (Hmim, C_4_H_6_N_2_), poly(3,4-ethylenedioxythiophene) polystyrene sulfonate (PEDOT:PSS), creatine, phosphate-buffered saline 7.4 (PBS 7.4) tablets, Potassium ferrocyanide trihydrate (K_4_Fe(CN)_6_·3H_2_O), potassium ferricyanide (K_3_Fe(CN)_6_), potassium chloride (KCl), and Polydimethylsiloxane (PDMS) were purchased from Merck, Taiwan. Deionized water (DI) was accessed from a Millipore Milli-Q plus 185 purification system with a resistivity of 18.2 MΩ.

### 2.2. Synthesis of ZIF-8 NPs

ZIF-8 NPs solutions were synthesized using Zn(NO_3_)_2_·6H_2_O as the metal precursor, 2 methyl imidazole (Hmim) as the organic precursor, and water as the solvent by mixing them in the molar ratio of 1:100:1238. First, Zn(NO_3_)_2_·6H_2_O and Hmim were individually added to 50 mL of DI water and were stirred by a magnetic stirrer for 30 min. the Zn salt solution was then added dropwise to the Hmim solution and stirred for 2 h at room temperature. The solution turned milky white gradually. The solution was centrifuged at 6000 rpm for 10 min and washed three times with pure DI water. The white-colored ZIF-8 powder was dried overnight in a drying oven at 90 °C. Finally, the ZIF-8 nanopowder was obtained and redispersed in ethanol. 

### 2.3. Device Fabrication

The ITO substrate was cleaned by ultrasonication consecutively in acetone and (IPA) for 5 min. The ITO was dried in an oven, and oxygen plasma was treated on the ITO for 5 min to make the surface hydrophilic. The ITO was cut into 2 cm × 1 cm pieces. PEDOT:PSS was drop-casted and dried on the ITO surface by a hot plate. The sample was immersed into the ZIF-NPs dispersion solution overnight to form ZIF-8 NPs thin film on the PEDOT:PSS/ITO film. The film was heated at 80 °C for solvent evaporation. A circular sensing window with a 1 mm wide opening was defined at one side of the film by automatically dispensing resin from a robotic dispenser. The portion of the sample other than the sensing window was covered by PDMS to resist the exposure of the analyte. 

### 2.4. Instrumentation for Physical and Electrochemical Characterization

The surface morphology of the sensing electrode was investigated by FESEM images using a JEOL JSM 7500F instrument with an operating accelerating voltage of 5 kV. The two-dimensional topology of the films was investigated by a Vecco D5000 AFM instrument using a tapping method with a Si probe. The crystalline structure and orientations were interpreted by XRD analysis using Rigaku D/MAX2000 with a copper (Cu) Kα (1.54176 Å). The XPS analysis to investigate the chemical composition and coordination was obtained by a PHI 5800 ESCA XPS system where monochromated aluminum (Al) Kα X-ray (1486.6 eV) with a spot diameter of 100 µm was used. The Gaussian–Lorentzian function was used for line contour fitting, and Shirley’s background subtraction was used for peak fitting.

**Scheme 1:**
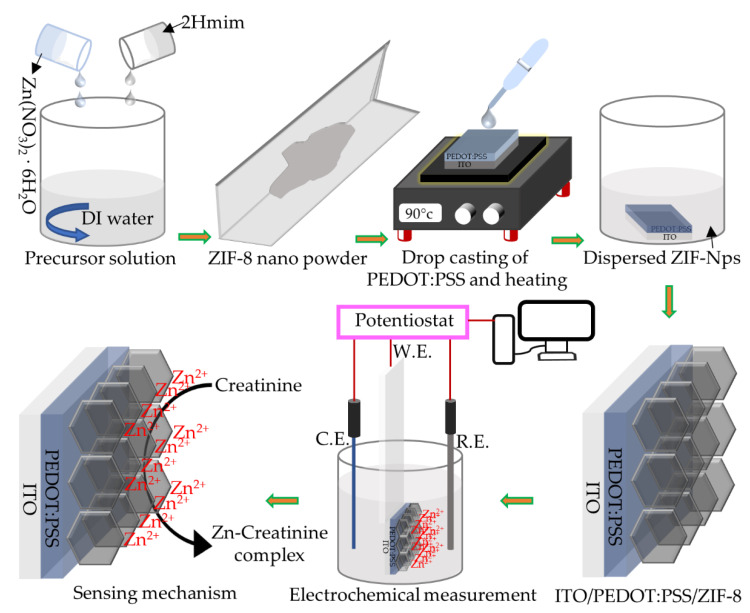
Schematic illustration presenting the fabrication of ZIF-8 NPs/PEDOT:PSS/ITO based creatinine sensors and their sensing mechanism.

The electrochemical characterization of the creatinine sensors was executed by a three-electrode system using palmsens4, an electrochemical workstation. The PSTrace 5.6 software was utilized to visualize the CV, DPV, and EIS spectra of the electrode. A platinum (Pt) wire and the silver/silver chloride (Ag/AgCl) filled with 3 M KCl were used as the counter electrode and reference electrode, whereas ZIF-8 NPs/PEDOT:PSS/ITO was used as the working electrode. The three electrodes were mounted equidistant on top of the beaker containing electrolyte fixed with the help of a PDMS block and were connected to the monitor through palmsens4 potentiostat. The CV and EIS measurements were performed in PBS 7.4 containing 1 mM [Fe(CN)6]^3−/4−^ and 0.1 M KCl. The redox properties of bare ITO, PEDOT:PSS/ITO electrode, ZIF-8 NPs/PEDOT:PSS/ITO electrode were compared by CV and EIS. The CV curves for different modification steps were scanned for a potential window of −0.8 V to 1 V at a scan rate of 50 mV/S. In the case of EIS measurement, the Nyquist curves were obtained by applying Randle’s equivalent circuit to fit the EIS spectra for a frequency ranging 1 Hz to 100 kHz for an AC potential of 5 mV with a DC potential of 0.5 V. Randle’s circuit consists of the electrolyte resistance (R_s_) in series with the parallelly connected double-layer capacitance (C_dl_) and the faradic reaction impedance. The faradic reaction is interpreted by combining the charge transfer resistance (R_ct_) and Warburg diffusion element (Z_w_). The values of R_s_, R_ct_, and C_dl_ were calibrated as 560 Ω, 10,000 Ω, and 33 nF, respectively. The sensing performance of the optimized ZIF-8 NPs/PEDOT:PSS/ITO electrode in different concentrations of creatinine (0.05 Mm–2.5 mM) added to PBS 7.4 containing 1 mM [Fe(CN)_6_]^3−/4−^ and 0.1 M KCl were determined by EIS measurement. The charge transfer resistance R_ct_ was considered the critical parameter for evaluating creatinine sensing. The limit of detection (LOD) of the sensor was estimated by the EIS calibration plot, a (ΔR_ct_/R_ct_(0)) × 100% vs. creatinine concentration plot, where ΔR_ct_ is the difference between R_ct_ of the analyte and the R_ct_ of the blank (R_ct_(0)). The selectivity of the creatinine sensor for creatinine (1 mM) compared to glucose (3 mM), urea (5 mM), and cystatin C (2 mM) was analyzed by chronoamperometry and EIS. The commercial applicability of the creatinine sensor was confirmed by measuring the sample in actual human serum containing different amounts of creatinine (0.05 mM, 1 mM, and 2.5 mM) and validated by calculating recovery rates compared to the performance in PBS 7.4. The ZIF-8 NPS/PEDOT:PSS/ITO electrode fabrication and its creatinine detection mechanism are described in Scheme 1.

## 3. Results

### 3.1. Morphological, Structural, and Stoichiometric Characterization

**Figure 1:**
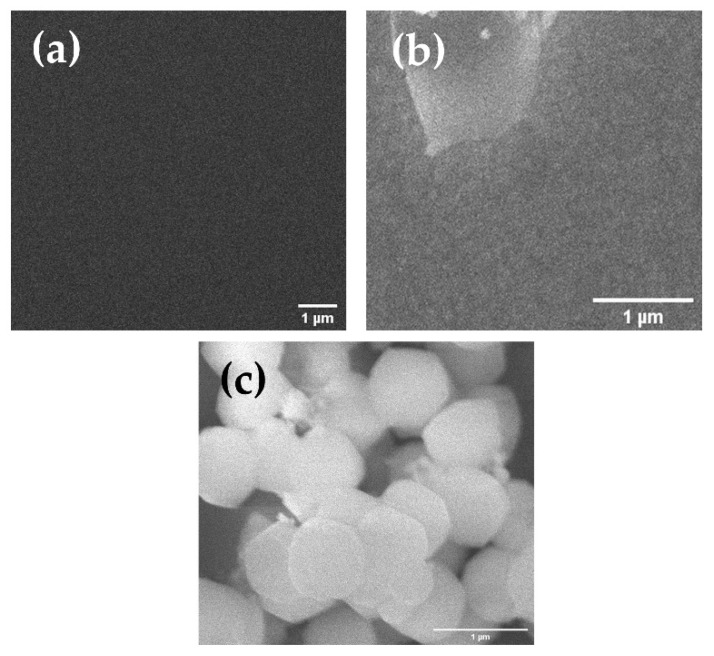
FESEM images for (**a**) bare ITO (**b**) PEDOT:PSS/ITO, and (**c**) ZIF-8 NPs/PEDOT:PSS/ITO.

To visualize the surface morphology of the bare ITO, PEDOT:PSS coated ITO, and ZIF-8 NPs/PEDOT:PSS/ITO film, FESEM images were analyzed. A flat surface was observed with no grains in the FESEM image of bare ITO depicted in Figure 1a, whereas a vague grain growth was noticed upon coating the ITO surface with PEDOT:PSS, evident from Figure 1b. Figure 1c shows the uniform distribution of polyhedral-shaped ZIF-8 NPs. The FESEM image of the densely packed ZIF-8 nanoparticles confirmed the lower degree of agglomeration with a distinct boundary of each particle. 

**Figure 2:**
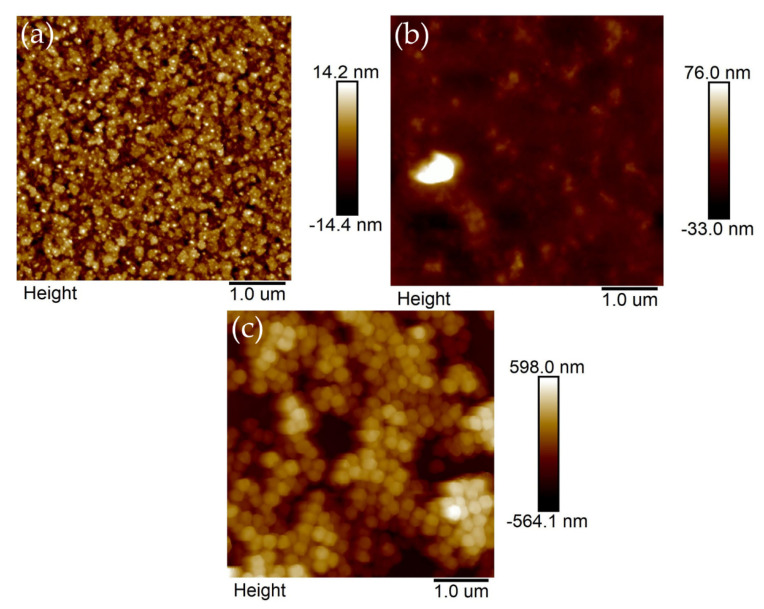
Two dimensional AFM images for (**a**) bare ITO (**b**) PEDOT:PSS/ITO, and (**c**) ZIF-8 NPs/PEDOT:PSS/ITO.

The topography of the bare ITO, PEDOT:PSS/ITO, and ZIF-8 NPs/PEDOT:PSS/ITO was examined by 2 dimensional AFM analysis in Figure 2a–c, respectively. The AFM image of bare ITO showed a vague microstructure with the root mean square (R.M.S.) surface roughness (R_q_) 4.14 nm. The topology of the ITO substrate noticeably improved upon functionalization with PEDOT:PSS. 

However, the formation of the ZIF-8 NPs was clearly visible from the AFM shown in Figure 2c. The morphology of ZIF-8 NPs/PEDOT:PSS/ITO film showed the uniform distribution of the polyhedron-shaped particles with a dense and smooth surface. The polygonal faces and sharp edges confirmed the formation of ZIF-8 nanostructure. There was no significant evidence of particle aggregation, which is favorable for the sensing mechanism. A high surface roughness R_q_ = 149 nm was achieved in ZIF-8 NPs/PEDOT:PSS/ITO film.

**Figure 3:**
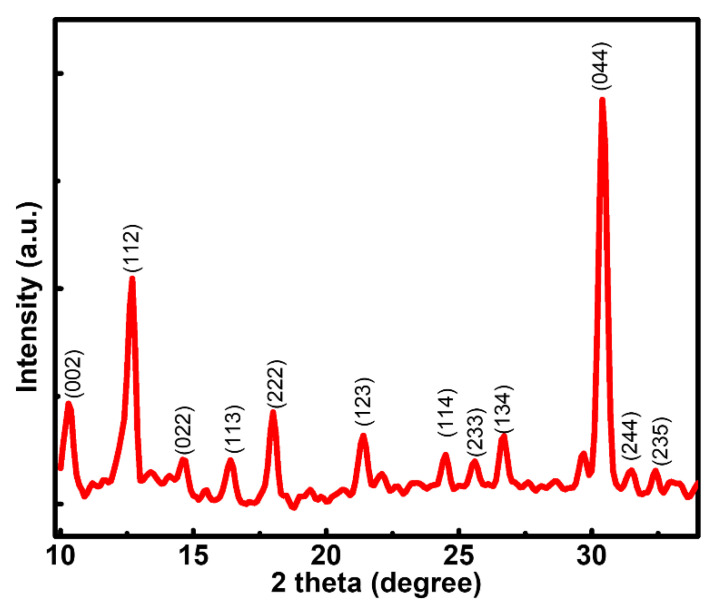
XRD spectra for ZIF-8 nanoparticles.

The crystalline structure and orientation were investigated by XRD analysis in Figure 3 for 2 thetas ranging from 0° to 35°. The spectra showed the presence of (002), (112), (022), (113), (222), (123), (114), (233), (134), (044), (244), and (235) planes. The high intensity of (112), (022), and (044) peaks confirmed the formation of ZIF-8 NPs. The results were similar to previously reported XRD in reference [17,18]. The prominent XRD peaks indicated well-crystalline ZIF-8 nanostructure, which is also evident from the AFM study. 

**Figure 4:**
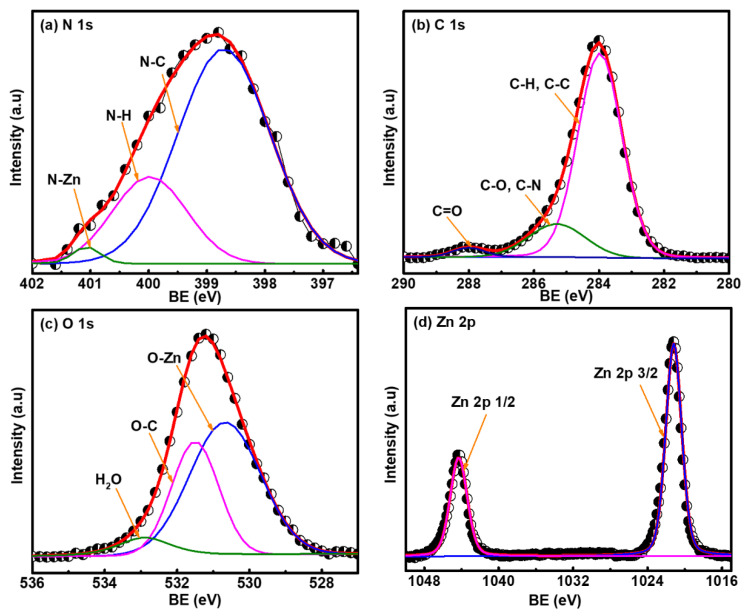
XPS of (**a**) N 1s (**b**) C 1s (**c**) O 1s, and (**d**) Zn 2p of ZIF-8 NPs.

The elemental composition and coordination elucidated by XPS analysis are depicted in Figure 4. As ZIF-8 NPs consist of Zn (inorganic) and 2-methyl imidazole (organic), the XPS analysis of nitrogen (N) 1s, carbon (C) 1s, oxygen (O) 1s, and Zn 2p illustrates the stoichiometry of the compound. In Figure 4a, the XPS of N 1s was deconvoluted into three different peaks at binding energies (BE) of 401.037 eV, 399.98 eV, and 398.71 eV representing the Zn-N, N-H, and N-C bonds, respectively. Primarily, the Zn-N bond represented the chemical interaction of the metal and organic components. 

The interaction of other atoms with carbon was investigated by analyzing the C 1s peak in Figure 4b. The C 1s spectra consisted of C=O, C-O/C-N, and C-H/C-C peaks at 287.98 eV, 285.31 eV, and 283.96 eV, respectively. The presence of Zn-O, Zn-OH, and C-O bonds was confirmed 530.66 eV, 532.93 eV, and 531.50 eV, respectively, in the XPS of O 1s shown in Figure 4c. The minimized peak intensities of Zn-OH determined the less amount of impurity present in ZIF-8. Moreover, the occurrence of Zn^2+^ oxidation of zinc in ZIF-8 was confirmed by the XPS of Zn 2p in Figure 4d. The spectral line consisted of the distinct doublets Zn 2p_3/2_ and Zn 2p_1/2_ located at 1021.23 eV and 1044.26 eV, respectively. The XPS result of this study was in good agreement with the previously reported data [19,20]. 

### 3.2. Electrochemical Characterization of the ZIF-8 NPs/PEDOT:PSS/ITO Sensing Electrode

**Figure 5:**
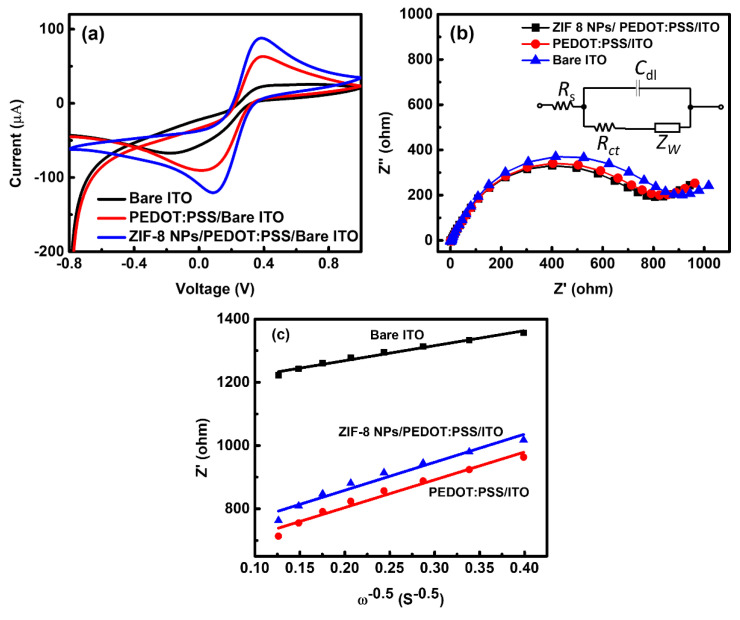
(**a**) CV characterization of the modification steps of ZIF-8 NPs/PEDOT:PSS/ITO electrode in PBS 7.4 containing 1 mM [Fe(CN)_6_]^3−/4−^ and 0.1 M KCl at a scan rate of 50 mV/S and (**b**) EIS characterization of the modification steps of ZIF-8 NPs/PEDOT:PSS/ITO electrode in PBS 7.4 containing 1 mM [Fe(CN)_6_]^3−/4−^ and 0.1 M KCl. The inset shows the equivalent Randle’s circuit. (**c**) Z’ vs. ω^−0.5^ plot obtained from Figure 5b for bare ITO, PEDOT:PSS/ITO, and ZIF-8 NPs/PEDOT:PSS/ITO electrodes.

Figure 5a showed the ITO modification steps by PEDOT:PSS followed by ZIF-8 NPs in CV measurement system. The CV curves for different modification steps were scanned for a potential window of −0.8 V to 1 V at a scan rate of 50 mV/S in PBS 7.4 containing 1 mM [Fe(CN)_6_]^3−/4−^ and 0.1 M KCl. The Fe^3+^/Fe^2+^ redox exchange occurred at the bare ITO electrode at the anodic voltage (E_pa_) of 0.40 V, and cathodic peak (E_pc_) −0.138 V. The diminished anodic current/cathodic current (I_pa_/I_pc_ = 34.86 µA/68.17 µA) and the broad separation potential (ΔE_p_) of 1.78 V signified the sluggish electron transfer of the bare ITO. The PEDOT:PSS coating on ITO increased the redox peaks I_pa_/I_pc_ = 88.89 µA/97.19 µA and minimized the ΔE_p_ to 0.35 V. Moreover, the almost equal value of I_pa_ and I_pc_ confirmed the electrochemical reversibility of the electrode. Finally, the ZIF-8 NPs/PEDOT:PSS/ITO electrode depicted sharper redox peaks I_pa_/I_pc_ = 124.46 µA/129.88 µA at E_pa_/E_pc_ = 0.38 V/0.09. The reduced value of ΔE_p_ (0.29) and steeper redox peaks confirmed optimized electrochemical properties of the ZIF-8 NPs/PEDOT:PSS/ITO electrode.

The variation in the electrochemical properties due to the modification steps of the electrode was also verified by EIS measurement. The Nyquist curves of bare ITO, PEDOT:PSS/ITO, and ZIF-8 NPs/PEDOT:PSS/ITO was obtained in Figure 5b by applying Randle’s equivalent circuit to fit the EIS spectra. Randle’s circuit consists of the electrolyte resistance in series with the parallelly connected double-layer capacitance (C_dl_) and the faradic reaction impedance. The faradic reaction is interpreted by combining the charge transfer resistance (R_ct_) and Warburg diffusion element (W). The Nyquist plots of different electrodes consist of a semicircular portion at high frequency, defining the redox reaction and the linear part represents the mass transfer. The charge transfer resistance (R_ct_), the radius of the semicircular region, was considered as the evaluation parameter of the redox exchange on the electrode. The value of R_ct_ of the bare ITO, PEDOT:PSS/ITO, and ZIF-8/PEDOT:PSS/ITO was obtained as 881.4 Ω, 820.2 Ω, and 800.3 Ω, respectively. The decrease in R_ct_ due to the modification of bare ITO by PEDOT:PSS followed by ZIF-8 NPs signified enhanced electrical conductivity consistent with the results of CV characterization. To obtain the diffusion coefficients of the bare ITO, PEDOT:PSS/ITO, and ZIF-8/PEDOT:PSS/ITO electrodes, the Warburg coefficients (σ_w_) were obtained as the slope of the Z’ vs. the reciprocal root of angular frequency (ω^−0.5^) plot as presented in Figure 5c. The values of σ_w_ for bare ITO, PEDOT:PSS/ITO, and ZIF-8/PEDOT:PSS/ITO electrodes were 474.12 ΩS^−0.5^, 878.19 ΩS^−0.5^, and 888.02 ΩS^−0.5^, respectively. The diffusion coefficients of the electrodes were calculated using Equation (1):

(1) $$D=0.5{\left(\frac{RT}{AF^{2}\sigma_{w}C}\right)}^{2}$$

where R is the universal gas constant (8.314 J mol^−1^ K^−1^), T represents the room temperature (298.5 K), A is the active electrode area, F is the Faraday’s constant (96500 C mol^−1^), and C is the molar concentration of Fe in the electrolyte. The estimated diffusion coefficients for bare ITO, PEDOT:PSS/ITO, and ZIF-8/PEDOT:PSS/ITO electrodes were 1 × 10^−17^ Ω cm^2^ S^−0.5^, 2.91 × 10^−18^ Ω cm^2^ S^−0.5^, and 2.85 × 10^−18^ Ω cm^2^ S^−0.5^, respectively. 

**Figure 6:**
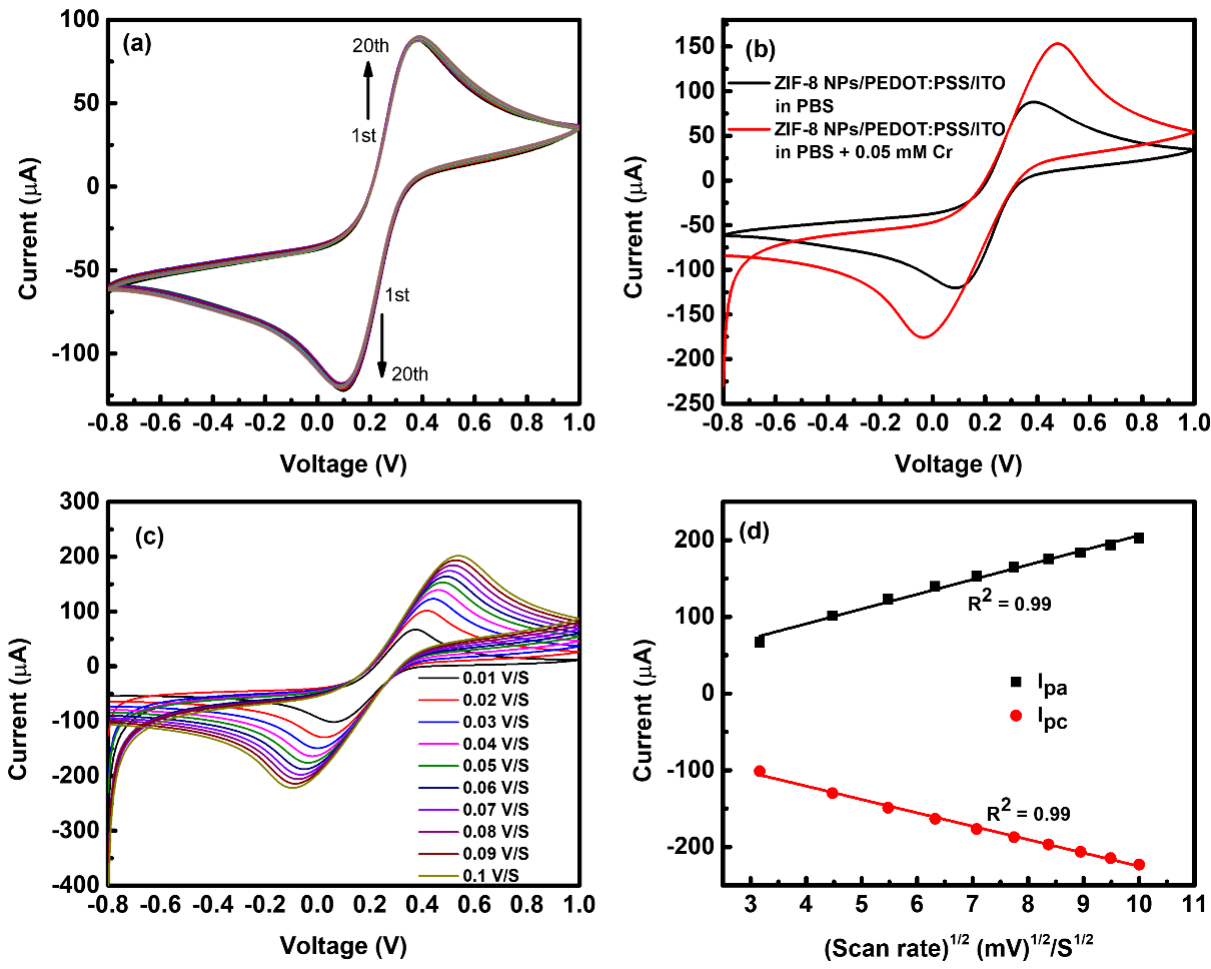
(**a**) CV for 20 cycles of ZIF-8 NPs/PEDOT:PSS/ITO electrode in PBS 7.4 containing 1 mM [Fe(CN)_6_]^3−/4−^ and 0.1 M KCl at a scan rate of 50 mV/S (**b**) comparison of CV curves for ZIF-8 NPs/PEDOT:PSS/ITO electrode in PBS 7.4 containing 1 mM [Fe(CN)_6_]^3−/4−^ and 0.1 M KCl with and without 0.05 mM of creatinine (**c**) CV of ZIF-8 NPs/PEDOT:PSS/ITO electrode for different scan rates (10 mV/S to 100 mV/S) in PBS 7.4 containing 1 mM [Fe(CN)_6_]^3−/4−^ and 0.1 M KCl containing 0.05 mM of creatinine (**d**) The plot of redox peak currents I_pa_ and I_pc_ vs. square roots of the scan rates.

The stability of the ZIF-8 NPs/PEDOT:PSS/ITO electrode was investigated by measuring the CV in PBS 7.4 containing 1 mM [Fe(CN)_6_]^3−/4−^ and 0.1 M KCl for repetitive 20 cycles. The CV curves of 20 successive cycles in Figure 6a demonstrated no significant changes in the peak currents signified the consistent redox exchange capability of the electrode. However, an inconsiderable change in peak current and ΔE_p_ occurred due to a hydrous layer formation on the electrode surface, creating a blocking layer for the electron transfer between the electrode and analyte. 

The creatinine sensing ability of the ZIF-8 NPs/PEDOT:PSS/ITO electrode was verified by measuring the CV of the electrode in pure PBS 7.4 and PBS 7.4 containing creatinine, as shown in Figure 6b. The CV of the electrode in PBS 7.4 containing creatinine showed higher redox currents (I_pa_/I_pc_ = 156.91 µA/168.67 µA), confirming the formation of a soluble Zn-creatinine complex form on the ZIF-8 sensing electrode. The nearly equal value of the two redox peaks signified the almost reversible change during Zn-creatinine interaction.

The effect of alteration in scan rate (10 mV/S to 100 mV/S) on the Zn-creatinine complex formation at ZIF-8 NPs/PEDOT:PSS/ITO was investigated in Figure 6c. The oxidation and reduction peak of the CVs were increased with the increasing scan rate. The redox voltage separation (ΔE_p_) was also enhanced upon increasing scan rate. This result indicates that the formation of Zn-creatinine soluble complex attributed to the charge-transfer phenomena increased with higher scan rates on the ZIF-8 NPs/PEDOT:PSS/ITO sensing electrode. The oxidation and reduction peak (I_pa_/I_pc_) ratio for all scan rates remained nearly unity, suggesting the almost reversible charge transfer kinetics due to creatinine chelating on the electrode. The redox peak currents I_pa_ and I_pc_ were plotted against the square roots of the scan rates in Figure 6d. The plot showed linearity of R^2^ = 0.99 and R^2^ = 0.99 for I_pa_ and I_pc_, respectively. The excellent linearity suggested the creatinine and ZIF-8 NPs/PEDOT:PSS/ITO interaction was highly adsorption controlled.

**Figure 7:**
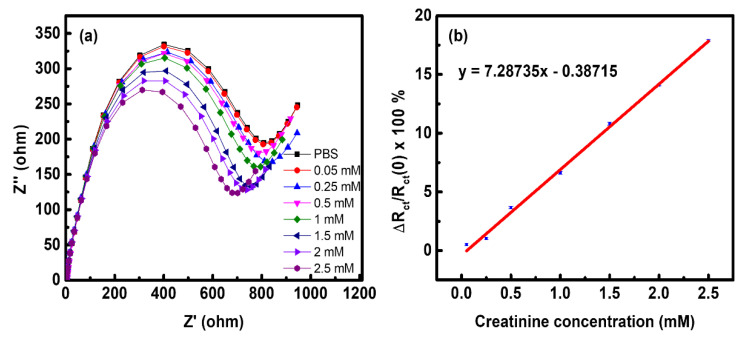
(**a**) EIS curves for ZIF-8 NPs/PEDOT:PSS/ITO electrode in different concentrations of creatinine (0.05 mM to 2.5 mM) (**b**) (ΔR_ct_/R_ct_(0)) × 100% vs. creatinine concentration plot.

As CV and EIS both are complementary electroanalytical tests to analyze the electrochemical double layer on the electrodes, the sensitivity of the ZIF-8 NPs/PEDOT:PSS/ITO based creatinine sensor was quantified by EIS measurement (Figure 7a) upon successive addition of creatinine concentration ranging from 0.05 mM to 2.5 mM. The Nyquist plots for different creatinine concentrations were measured in a frequency ranging from 100 kHz to 1 Hz in PBS containing [Fe(CN)_6_]^3−/4−^ and 0.1 M KCl. The EIS of all the concentrations were obtained at a fixed potential of 0.5 V, the oxidation peak potential of ZIF-8 NPs/PEDOT:PSS/ITO sensing electrode measured in PBS 7.4 containing 1 mM [Fe(CN)_6_]^3−/4−^ and 0.1 M KCl with 0.05 mM creatinine, was determined from the CV characteristics (Figure 6b). The curvature of the semicircle related to the charge transfer resistance R_ct_ decreased with increasing creatinine concentration, indicating the enhanced oxidation on the electrode. The ZIF-8 NPs/PEDOT:PSS/ITO based creatinine sensor performance was assessed by the EIS calibration curve (Figure 7b), a (ΔR_ct_/R_ct_(0)) × 100% vs. creatinine concentration plot, where ΔR_ct_ is the difference between R_ct_ of the analyte and the R_ct_ of the blank (R_ct_(0)). The slope of the calibration plot was obtained from the equation of the fitted line given by y = 7.28735x−0.38715 with a correlation coefficient of R^2^ = 0.99. The limit of detection (LOD) was estimated from the calibration plot given by the well-known equation LOD = k × SD/b, where K represents the signal to noise ratio (S/N) considered as 3:1, SD is the standard deviation in PBS calculated for three replications (*n* = 3), and b is the slope of the calibration plot. The estimated LOD for the ZIF-8 NPs/PEDOT:PSS/ITO electrode was 0.03 mM in the linear range 0.05 mM to 2.5 mM, which was relatively lower than other reported LODs, tabulated in Table 1.

**Figure 8:**
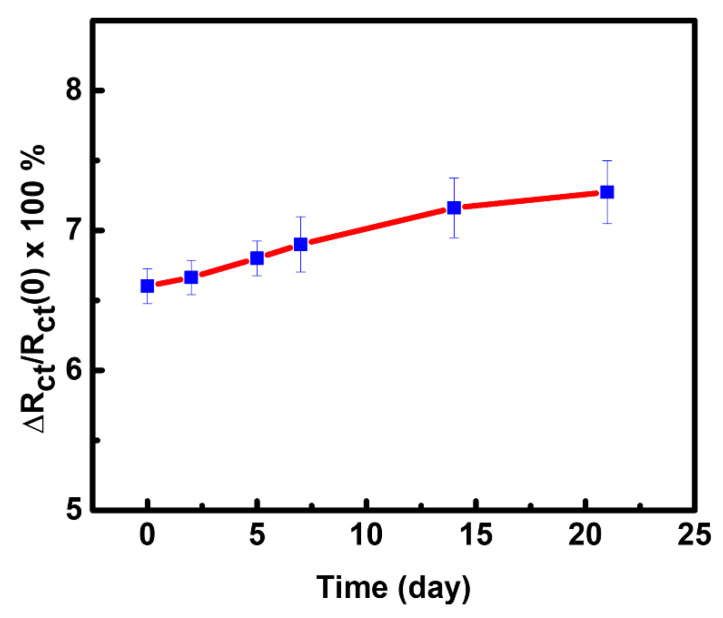
Long-term (21 days) durability test of ZIF-8 NPs/PEDOT:PSS/ITO based creatinine sensor in 1 mM creatinine solution obtained from EIS measurement.

To examine the durability of the ZIF-8 NPs/PEDOT:PSS/ITO based creatinine sensor, the durability test was performed for 21 days, as shown in Figure 8. The EIS measurement of the creatinine sensor was recorded in 1 M creatinine containing electrolyte for day 0, day 2, day 5, day 7, day 14, and day 21. The relative percentage change in R_ct_ was preserved up to 90%, which confirmed the excellent durability of the sensor for 21 days. 

**Figure 9:**
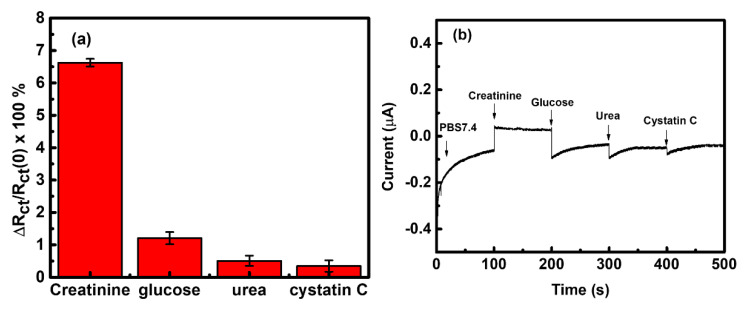
(**a**) Selectivity analysis of ZIF-8 NPs/PEDOT:PSS/ITO sensing electrode for creatinine compared to glucose (3 mM), urea (5 mM), and cystatin C (2 mM) (**b**) Chronoamperometric characterization obtained for the creatinine (1 mM) attachment at ZIF-8 NPs/PEDOT:PSS/ITO electrode with the successive addition of glucose (3 mM), urea (5 mM), and cystatin C (2 mM).

The selectivity of the ZIF-8 NPs/PEDOT:PSS/ITO creatinine sensor towards creatinine (1mM) compared to relatively relevant markers, such as glucose (3 mM), urea (5 mM), and cystatin C (2 mM), was performed by analyzing their corresponding EIS spectra (Figure 9a) and amperometric characterization (Figure 9b). For EIS analysis, the changes in (ΔRct/Rct(0)) × 100% for each biomolecule were compared by a bar graph presented in Figure 7. The concentrations of each of the biomolecules were relevant to their clinical range in actual serum. The (ΔRct/Rct(0)) × 100% value of creatinine was 5–17 times higher than other interfering molecules. Hence, we can confirm that the ZIF-8 NPs/PEDOT:PSS/ITO sensing electrode was highly selective to its target molecule, creatinine. In the case of amperometric characterization, creatinine, glucose, urea, and cystatin c were added to PBS after every 100 s. A significant increment in current was observed after adding creatinine, whereas minor current alterations were observed after adding other biomolecules (glucose, urea, cystatin c). 

**Figure 10:**
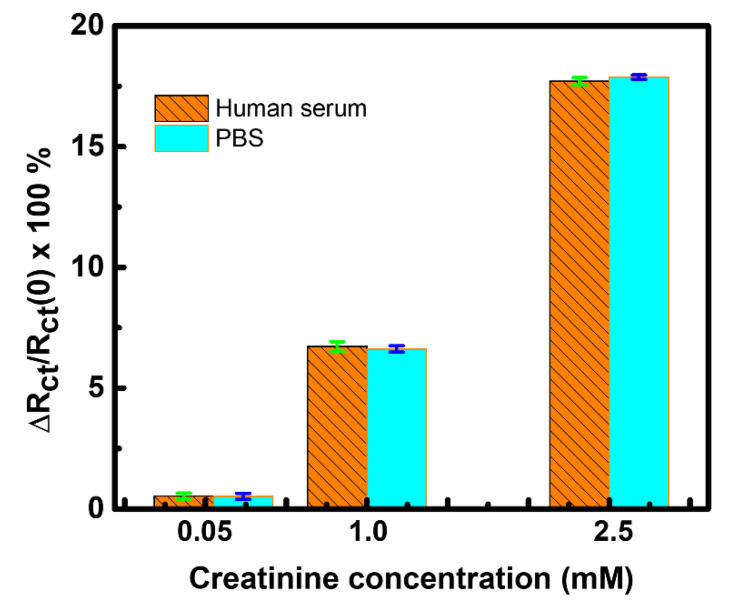
Comparison bar graph of creatinine detection by ZIF-8 NPs/PEDOT:PSS/ITO in PBS and human serum.

The practical applicability of the ZIF-8 NPs/PEDOT:PSS/ITO creatinine sensor was investigated by applying the electrode in actual human serum. The human serum was spiked with three different creatinine concentrations such as 0.05 mM, 1 mM, and 2.5 mM. A comparison bar graph showing the (ΔR_ct_/R_ct(0)_) × 100% value of each concentration in PBS and human serum is depicted in Figure 10. The amount of creatinine detected by the electrode in the human serum is very close to the concentration measured in PBS and the recovery percentage varied in the range 101–102.5%. As a deduction, the electrode can be considered an efficient biosensor that can detect the existence of creatinine in an actual human serum sample.

## 4. Conclusions

The purpose of this study was to develop a ZIF-8 NPs/PEDOT:PSS/ITO based sensing electrode for the non-enzymatic electrochemical creatinine sensor. The ZIF-8 NPs were synthesized by mixing zinc and Hmim precursors with DI water. The FESEM, AFM, and XRD analysis confirmed the polyhedral ZIF-8 nanoparticle formation and crystallinity, respectively, whereas the elemental composition and interatomic coordination were elucidated by XPS analysis. The electrochemical behavior of the electrode was determined by the CV, and EIS analysis. The LOD of the sensor was determined by EIS measurement. The sensor obtained a sufficiently low limit of detection (LOD) of 0.03 mM in a clinically acceptable linear range (0.05 mM–2.5 mM). The selectivity analysis of the sensor showed high selectivity of the sensor for creatinine compared to other interfering biomolecules. The sensing performance of the creatinine sensor was verified in the actual human serum, which showed excellent recovery rates. The excellent sensing performance of the ZIF-8 based creatinine sensor proved its potential to recognize other kidney-related biomolecules. 

## Figures and Tables

**Table 1:** Comparison of ZIF-8 NPs based creatinine sensor with other existing creatinine sensors.

Sensing Material	Method of Detection	Measurement Technique	LOD (µM)	Linear Range
Β-cyclodextrin incorporated poly-3,4-ethylene dioxythiophene mofified glassy carbon electrode [21]	Non-enzymatic	Potentiometric	50	100–100,000
PANi [22]	Enzymatic	Amperometric	50	50–1000
Poly venyle chloride [23]	Enzymatic	Potentiometric	44–88	88–4400
Paper absorbed Fe (III) ions [24] Microfluidic cartridge [25]	Non-enzymatic Enzymatic	Amperometric Colorimetric	43 60	100–6500 67.2–1768
This work	Non-enzymatic	Impedemetric	30	50–2500

## Data Availability

The data used to support the findings of this study are available from the corresponding author upon request.
